# Variation in the FFAR1 Gene Modifies BMI, Body Composition and Beta-Cell Function in Overweight Subjects: An Exploratory Analysis

**DOI:** 10.1371/journal.pone.0019146

**Published:** 2011-04-28

**Authors:** Celia G. Walker, Louise Goff, Les J. Bluck, Bruce A. Griffin, Susan A. Jebb, Julie A. Lovegrove, Thomas A. B. Sanders, Gary S. Frost, on behalf of the RISCK Study Group

**Affiliations:** 1 Elsie Widdowson Laboratory, MRC Human Nutrition Research, Cambridge, United Kingdom; 2 Nutrition and Dietetic Research Group, Imperial College London, London, United Kingdom; 3 Faculty of Health and Medical Sciences, University of Surrey, Guildford, United Kingdom; 4 Hugh Sinclair Unit of Human Nutrition and Institute for Cardiovascular and Metabolic Nutrition (ICMR), University of Reading, Reading, United Kingdom; 5 Nutritional Sciences Division, Kings College, University of London, London, United Kingdom; Mayo Clinic College of Medicine, United States of America

## Abstract

**Background:**

FFAR1 receptor is a long chain fatty acid G-protein coupled receptor which is expressed widely, but found in high density in the pancreas and central nervous system. It has been suggested that FFAR1 may play a role in insulin sensitivity, lipotoxicity and is associated with type 2 diabetes. Here we investigate the effect of three common SNPs of *FFAR1* (rs2301151; rs16970264; rs1573611) on pancreatic function, BMI, body composition and plasma lipids.

**Methodology/Principal Findings:**

For this enquiry we used the baseline RISCK data, which provides a cohort of overweight subjects at increased cardiometabolic risk with detailed phenotyping. The key findings were SNPs of the *FFAR1* gene region were associated with differences in body composition and lipids, and the effects of the 3 SNPs combined were cumulative on BMI, body composition and total cholesterol. The effects on BMI and body fat were predominantly mediated by rs1573611 (1.06 kg/m^2^ higher (P = 0.009) BMI and 1.53% higher (P = 0.002) body fat per C allele). Differences in plasma lipids were also associated with the BMI-increasing allele of rs2301151 including higher total cholesterol (0.2 mmol/L per G allele, P = 0.01) and with the variant A allele of rs16970264 associated with lower total (0.3 mmol/L, P = 0.02) and LDL (0.2 mmol/L, P<0.05) cholesterol, but also with lower HDL-cholesterol (0.09 mmol/L, P<0.05) although the difference was not apparent when controlling for multiple testing. There were no statistically significant effects of the three SNPs on insulin sensitivity or beta cell function. However accumulated risk allele showed a lower beta cell function on increasing plasma fatty acids with a carbon chain greater than six.

**Conclusions/Significance:**

Differences in body composition and lipids associated with common SNPs in the *FFAR1* gene were apparently not mediated by changes in insulin sensitivity or beta-cell function.

## Introduction

Body mass index is an established independent risk factor for the development of type 2 diabetes (T2D). Non-esterified free fatty acids (NEFA) have been implicated in the development of T2D through effects on insulin release and the development of the metabolic syndrome. The free fatty acid receptor FFAR1 (GPR40 – G-protein-coupled receptor 40) was the first gene product identified to act as an extracellular membrane receptor for FFAs [1]. It is located in the 19q13.1 chromosomal region, which has been linked to T2D and T2D-related phenotypes, in several genome-wide scans [2], [3] and is expressed widely in the pancreas, central nervous system (CNS) and adipocytes, particularly omental adipocytes [1]. Recent *in vitro* investigations have shown FFAR1 to be activated in pancreatic beta cells by medium- to long-chain FFAs as well as by thiazolidinediones (Rosiglitazone and MCC-555), causing elevated Ca^2+^ concentrations and subsequent promotion of insulin secretion. Furthermore, mice with over-expression of Ffarr1 show impaired beta cell function and develop diabetes [4], whereas disruption of the gene reduces FFA-stimulated insulin release and, possibly protects from diabetes [5]. Recently two papers have reported that a number of SNPs in the *FFAR1* gene mediate effects on insulin secretion, in particular in response to FFA [6], [7]. The role of FFAR1 in the CNS is not known, but it is hypothesised that this may be a mechanism by which FFAs are involved in the hypothalamic regulation of metabolism and its expression in omental adipocytes implicates it in the development of the metabolic syndrome [5]. It has been suggested more recently that FFAR1 plays a role in the taste perception of fatty acids, but this is controversial and needs substantiating [8]. Here we report the relationship between three common *FFAR1* SNPs, with BMI, body composition, pancreatic function, insulin sensitivity and plasma lipids, in a cohort of overweight subjects identified to be at increased cardiometabolic risk.

## Methods

### Cohort

For this enquiry we used the RISCK study cohort. The RISCK study has been described in detail elsewhere [9]. In brief, the RISCK study was a randomized, controlled, parallel trial performed in free-living participants at 5 U.K. centres (University of Reading, Imperial College London, Kings College London, University of Surrey, and the Medical Research Council Human Nutrition Research [MRC-HNR]). A total of 720 participants were recruited, selected on the basis of their increased risk for the metabolic syndrome using a study-specific scoring system. All participants followed a 4-wk run-in period during which they were prescribed a high-saturated fat/high-glycemic index (HS/HGI) “reference” diet before being randomised to the reference diet or one of four isoenergetic dietary interventions to lower saturated fat. The main outcome was a measure of insulin sensitivity with secondary outcomes, including a range of cardiovascular risk markers.

At screening a fasting blood sample was taken and used to measure fasting lipids including total cholesterol; HDL-cholesterol; triglycerides and non-esterified fatty acids (NEFA). LDL-cholesterol was derived from the Friedwald equation [9]. Anthropometry was measured by standard procedures, and body composition by bioelectrical impedance analysis (BIA) see Jebb *et al.* for details [9]. Insulin sensitivity and beta-cell function were determined by intravenous glucose tolerance test (IVGTT). Insulin sensitivity (Si) and glucose effectiveness (Sg) were estimated using the MINMOD Millennium programme (Version 6.02). The area under the plasma insulin curve up to 19 minutes was computed as an indicator of endogenous insulin secretion (AIRg). The disposition index (DI), a measure of the ability of the beta-cells to compensate for insulin resistance, was calculated from AIRg and Si values [10]. For the purpose of this cross-sectional analysis we investigated the effect of three known common *FFAR1* SNPs on BMI, body composition and fasting lipid measures, at entry into the study, and on insulin sensitivity and beta-cell function following 4 weeks of a “reference” high saturated fat, run-in diet.

### SNP selection and genotyping

SNPs in the *FFAR1* gene region were identified using SNPSelector set for gene SNPs by gene name (http://snpselector.duhs.duke.edu/hqsnp36.html) and cross-checked with information in other databases (Genecards http://www.genecards.org/index.shtml; Entrez-SNP http://www.ncbi.nlm.nih.gov/snp). Due to the small population of the study, we concentrated on common allele variants, and for that reason SNPs with a reported Caucasian minor allele frequency (MAF) of <5% were excluded, and only SNPs in the HapMap were investigated. A resulting 3 SNPs were genotyped for *FFAR1* (rs2301151; rs16970264; rs1573611). A further SNP in *FFAR1* (rs1978013) which was previously associated with beta-cell function [7] was also genotyped. Selected SNPs were tested for linkage disequilibrium with Haploview Version 4.2 software (http://www.broadinstitute.org/haploview/haploview using the Hapmap download format, version 3, release R2) and using information from previous studies [7] none of the SNPs were in significant LD (r^2^<5% rs2301151 with rs1573611; r^2^<5% rs2301151 with rs16970264; r^2^ 1% rs1573611 with rs16970264 see **Figure S1**).

DNA was available for 530 participants of the RISCK study. Genotyping was performed by KBioscience using the KASPar genotyping system (Hoddesden, Herts, UK). All SNPs were successfully genotyped with a call rate >97%. Results could not be obtained for 15 participants due to genotyping failure rate in >1 SNP. Deviations from Hardy-Weinburg equilibrium were tested and one SNP (rs1978013) deviated significantly (P<0.001) and was excluded from further analysis. A further three attempts to redesign primers for genotyping this SNP, residing in a region of high homology with surrounding regions in this gene cluster, were unsuccessful.

The RISCK subjects, for which there was DNA available, consisted of an ethnic mix (81% White; 9% S-SE Asian, 7% Black African, 3% other). The allele frequency of all SNPs studied varied significantly by ethnicity, therefore this analysis was restricted to the Caucasian European subjects only, which represented the predominant group (n = 405).

### Effect of number of FFAR1 risk alleles carried with change in plasma fatty acid level on metabolic outcome

The RISCK study has been analysed for plasma fatty acid profiles as an independent assessment of dietary change [1]. The fatty acids greater than C-6 are agonists for FFAR1 receptor, recent evidence in-vitro suggest that n-3 fatty acids stimulate the greatest response activation causing a greatest rise in intracellular calcium [1]. To examine the impact of change in plasma fatty acids greater than C-6 and DHA and EPA on metabolic outcome we analysed those subjects that has a positive change in plasma levels of the fatty acids between the baseline visit and end visit at week 24 giving a cohort of 280 of the volunteers.

### Statistical analysis

Data were tested for normal distribution and log-transformed for analysis where appropriate, as indicated in tables. Non-normally distributed data were presented as the geometric mean ± 95% confidence intervals. The cohort was separated by genotype and the mean value for each trait presented according to an additive model for each SNP. Each SNP was scored as 0,1,2 according to the number of BMI-increasing alleles carried for rs2301151 and rs1573611. The SNP rs16970264 had no association with BMI and was therefore scored 0,1,2 based on the number of total cholesterol increasing alleles carried. The BMI- or total cholesterol- increasing allele is referred to as the “risk allele”. The association between *FFAR1* SNPs and measures of body composition, insulin sensitivity and lipids were carried out using linear regression analysis. Age and gender were included in all models as covariates. For insulin sensitivity and lipid measures the associations were further adjusted for BMI. The effect of genotype on trait was then examined as dominant and recessive models based on the BMI or total cholesterol-increasing risk alleles. The mean of each trait divided by genotype is presented as the recessive and dominant models. The association of risk alleles according to the dominant and recessive models were also tested by linear regression. For the dominant model, a score of 0 was assigned for no risk alleles and 1 for presence (heterozygous or homozygous) of risk alleles, and for the recessive model, a score of 1 was assigned for homozygous for the risk allele and 0 for the others. We used the sum of risk alleles from the dominant models, as the numbers of the rare homozygous genotype were too low to analyse as single genotypes. In the analysis to assess the impact of dietary change metabolic change we used the change in plasma fatty acid profiles, an independent assessment of dietary intake. Uncorrected P-values are presented, however to account for multiple comparisons, we used the False Discovery Rate controlling procedure (q* = 0.05) of Benjamini and Hochburg with BMI, insulin-sensitivity and lipid-related traits treated as different families of hypotheses [11]. It is indicated where P-values satisfied the calculated FDR constraints.

## Results

### Subject characteristics

The allele frequency, gender and age distribution of the subjects of white ethnic origin included in this study, are shown stratified by genotype for the three SNPs in **Table 1**. There were no significant differences in age or gender distributions between the genotypes of any of the three *FFAR1* SNPs analysed (**Table 1**). There was no significant difference in metabolic syndrome or CVD risk, according to the study-specific scoring system) between the genotypes of any of the three *FFAR1* SNPs.

**Table 1:** Characteristics of the study cohort by genotype for three SNPs of *FFAR1*.

	rs2301151				rs1573611				rs16970264			
SNP Type/location	Non-synonymous				Near gene 3′				Near gene 5′			
	AA	AG	GG	*P*	CC	TC	TT	*P*	AA	GA	GG	*P*
*n*	233	156	16		247	138	20		3	47	350	
Genotype frequency	0.58	0.38	0.04		0.61	0.34	0.05		0.01	0.12	0.87	
Minor allele F	0.23		0.22		0.07							
Age	52.4 (9.6)	54.4 (10.3)	52.4 (11.2)	0.16	53.6 (10.2)	52.6 (9.5)	54.4 (10.4)	0.56	56.7 (5.13)	54.4 (8.9)	53.0 (10.1)	0.56
Gender (M%/F%)	43/57	42/58	19/81	0.16	40/60	44/56	50/50	0.89	67/33	40/60	41/59	0.91
MS score	6 [5,7]	6 [5,8]	6 [5,8]	0.26	6 [5,8]	6 [5,8]	6 [4,6.5]	0.42	7 [6,8]	7 [5,8]	6 [5,7]	0.09
CVD score	2 [1,4]	2 [1,5]	1 [1,3]	0.35	2 [1,5]	2 [1,4]	4 [1,7]	0.43	2 [1,9]	2 [1,5]	2 [1,4]	0.83

The data are presented as mean (SD) for age, proportion (%) for gender and median [IQR] for metabolic syndrome (MS) and cardiovascular disease (CVD) risk score for each genotype of the three SNPs of *FFAR1* which were investigated. Differences (P) in characteristics between genotypes are indicated. The MS and CVD risk score were study specific see Jebb *et al.* [9]. SNP location data from the NCBI-SNP database.

### Effect of FFAR1 polymorphisms on measures of body mass and composition

Carriage of the G allele of rs2301151 was associated with a higher body fat (%) of 1.11% per allele (P = 0.03) when assessed as the additive model (**Table 2**) and 1.39% higher per risk allele (P = 0.02) when assessed as the dominant model (**Table S1**), accounting for age and gender, although these associations were not statistically significant when accounting for multiple comparisons by FDR procedure. Carriage of the C allele of rs1573611 was associated with a higher BMI, body fat (%) and waist circumference when examined as the dominant model (**Table S1**), and with BMI and body fat (%) as an additive model (**Table 2**). The associations with BMI and body fat (%) but not waist circumference were statistically significant when accounting for multiple comparisons. There was a significant interaction between rs1573611 and gender for waist circumference when examined as the additive (P = 0.03) and the recessive (P = 0.05) models. The effect of SNP on waist circumference was only significant in females (effect = 3.27±1.12 cm higher per C allele, P = 0.02).

**Table 2:** BMI, waist circumference and body fat by genotype for three SNPs of *FFAR1*.

	rs2301151					rs1573611					rs16970264				
				Difference	P				Difference	P				Difference	P
	AA	AG	GG			TT	TC	CC			AA	AG	GG		
***BMI (kg/m^2^)***															
mean	28.71	29.41	30.01	0.67	0.11	25.61	28.86	29.31	1.06	**0.009**	28.53	29.53	28.85	0.55	0.41
SE	0.32	0.35	1.67			0.85	0.41	0.31			2.25	0.60	0.26		
n	233	156	16			20	138	247			3	47	350		
***Waist circumference (cm)***															
mean	98.60	98.57	98.09	0.27	0.79	93.53	98.89	98.53	1.29	0.19	100.23	100.57	97.95	2.09	0.20
SE	0.85	0.95	2.72			3.02	1.09	0.76			4.41	1.68	0.66		
n	233	156	16			20	138	247			3	47	350		
***Body fat (%)***															
mean	33.19	34.77	37.34	1.11	0.03	28.05	33.30	34.68	1.53	**0.002**	28.50	35.02	33.88	−0.58	0.48
SE	0.56	0.69	1.99			1.52	0.71	0.55			3.70	1.31	0.46		
n	228	155	16			20	136	244			3	47	345		

Data are presented as mean, SEM, n stratified by genotype for each of the three SNPs. For each genotype the risk allele was defined as the BMI-increasing allele and the data are presented as the additive model. The differences in trait by genotype were assessed by linear regression analysis coding the number of risk alleles as 0,1,2. The P-value for the regression is presented and is in bold when reaching significance by the FDR-controlling procedure q* = 0.05. The recessive and dominant models defined according to the risk allele are shown in **Supplementary Table S1**.

There was no evidence of a SNP-gender interaction for rs2301151 or rs16970264 for any of the variables.

**Figure 1:**
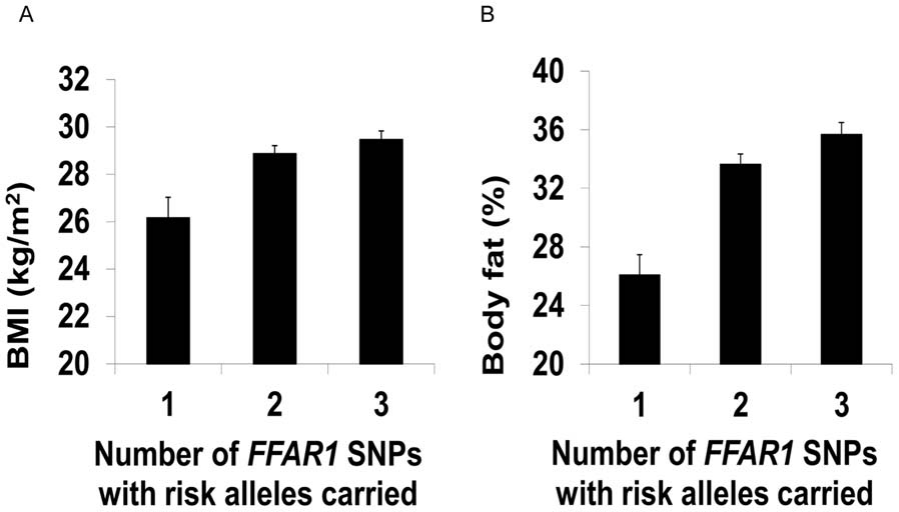
The cumulative effect of carrying risk alleles in three SNPs of *FFAR1* on BMI and body fat. Data presented are mean ± SEM of BMI (**Panel a**) and body fat (**Panel b**) for individuals according to the number of SNPs for which risk alleles were present. Each SNP (rs2301151; rs1573611; rs16970264) was scored 0 or 1 based on the presence of risk alleles (dominant model). The presence of risk alleles for each SNP was summed. All individuals had at least 1 SNP with risk alleles present. There was a cumulative effect on BMI of 1.04±0.41 kg/m2 per SNP, P = 0.01 and of body fat (%) of 1.75±0.6% per SNP, P = 0.001 assessed by linear regression analysis with age and gender as covariates. The effects were statistically significant when accounting for multiple comparisons by the Benjamini and Hochberg False Discovery Rate procedure with q* = 0.05.

There was a cumulative effect of the number of SNPs of *FFAR1* for which risk alleles were carried, on BMI and body fat (**Figure 1**); for an increasing number of SNPs, where at least one risk allele was carried, there was a higher BMI (effect = 1.04±0.41 kg/m2 per SNP, P = 0.01) and higher body fat-% (effect = 1.75±0.6% per SNP, P = 0.001). These effects were significant when accounting for multiple comparisons. There was no significant SNP×SNP interaction effect examined as either a two-way or three-way interaction using the additive model. There was no evidence of an effect of plasma fatty acid profile integrating with the cumulative number of risk alleles carried to have a significant effect on change in or final BMI, waist measurement and body fat content.

### Effect of FFAR1 polymorphisms on measures of insulin sensitivity

There was a nominally significant (unadjusted) association of the G allele of rs2301151 with a ∼73% higher disposition index (DI) and a trend (P = 0.06) towards a ∼52% higher endogenous (1st phase/acute) insulin release (AIRg) when assessed as the recessive model only, accounting for age, gender and BMI (**Table S2**). There was a nominally significant (unadjusted) dominant effect of the C allele of rs1573611 to be associated with lower fasting plasma glucose (−0.31 mmol/L, P = 0.02) (**Table S2**) There were no other effects on measures of insulin-sensitivity or beta-cell function, and none of these nominal associations reached significance when accounting for multiple comparisons by FDR procedure. There was no significant effect of rs16970264 on any measures of insulin-sensitivity or beta-cell function (**Table 3**). There was no evidence of gender-SNP interactions for any of the three SNPs on any measures of insulin-sensitivity or beta-cell function. There was no evidence of a cumulative effect of carrying risk alleles in multiple SNPs, nor was there any evidence of a two- or three-way interaction effect of the SNPs using the additive model (data not shown). There was a cumulative effect of carrying risk alleles in multiple SNPs with change in plasma fatty acid with AIRg measurement taken at 24 weeks. With the AIRg decreasing with the accumulated risk alleles (1 risk allele: 705.3±135 mL.µU^−1^.min^−1^, 2 risk alleles: 514.2±39 mL.µU^−1^.min^−1^, 3 risk alleles: 456.4±37 mL.µU^−1^.min^−1^ p<0.03, uncorrected). There was no other cumulative effect of risk alleles on insulin sensitivity.

**Table 3:** Measures of insulin sensitivity and beta-cell function by genotype for three SNPs of *FFAR1*.

	rs2301151					rs1573611					rs16970264				
				Difference	P				Difference	P				Difference	P
	AA	AG	GG			TT	TC	CC			AA	AG	GG		
***Fasting glucose (mmol/L)***															
mean	5.46	5.47	5.58	0.01	0.91	5.78	5.45	5.47	−0.07	0.18	3.57	5.52	5.47	−0.02	0.78
SE	0.04	0.05	0.52			0.13	0.06	0.04			0.20	0.08	0.03		
n	233	155	16			20	137	247			3	47	349		
***Fasting insulin (pmol/L)***															
Geometric mean	56.8	60.1	60.5	1.03	0.44	55.6	56.6	59.3	1.00	0.91	53.0	59.9	57.4	0.90	0.11
95% CI	[53.3, 64.4]	[56.1, 64.4]	[48.6, 75.4]			[46.9, 65.9]	[52.2, 61.4]	[55.9, 62.9]			[3.22, 873]	[57.8, 74.1]	[54.7, 60.2]		
n	227	153	13			20	135	238			3	45	340		
***Si (×10−4 mL.µU^−1^.min^−1^)***															
Geometric mean	2.81	2.69	3.20	1.03	0.49	3.02	2.74	2.80	1.04	0.36	1.68	2.53	2.84	1.15	0.09
95% CI	[2.60, 3.05]	[2.43, 2.97]	[2.74, 3.74]			[2.29, 3.98]	[2.44, 3.07]	[2.61, 3.00]			[0.00, 904]	[2.11, 3.03]	[2.66, 3.02]		
n	211	146	14			17	125	230			2	43	322		
***AIRg (mL.µU^−1^.min^−1^)***															
Geometric mean	443	319	521	1.01	0.73	255	354	335	1.01	0.83	997	320	337	0.96	0.42
95% CI	[403, 483]	[277, 368]	[398, 681]			[155, 418]	[311, 403]	[299, 374]			[0.51, 9185]	[241, 424]	[309, 369]		
n	211	146	14			17	125	230			2	43	322		
***Disposition Index (Arbitrary Units)***															
Geometric mean	972	864	1667	1.06	0.27	768	984	933	1.04	0.7	1673	808	960	1.08	0.51
95% CI	[864, 1093]	[737, 1012]	[1236, 2249]			[440, 1342]	[843, 1149]	[829, 1050]			[462, 6057]	[598, 1092]	[598, 1093]		
n	211	146	14			17	125	230			2	43	322		
***Sg (×10^−3^/min)***															
mean	17.21	17.94	20.67	1.23	0.18	20.32	18.04	17.06	−1.17	0.20	9.75	17.84	17.60	0.37	0.81
SE	0.42	1.15	1.76			2.56	1.24	0.45			3.96	1.63	0.55		
n	211	146	14			17	125	230			2	43	322		

Data are presented as mean, SEM, n (glucose, Sg) or geometric mean, 95% confidence intervals, n (insulin, Si, AIRg, DI) stratified by genotype for each of the three SNPs. For each genotype the risk allele was defined as the BMI-increasing allele and the data are presented as the additive model. The differences in trait by genotype were assessed by linear regression analysis coding the number of risk alleles as 0,1,2. Data for insulin, Si, AIRg and disposition index were logged for regression analysis. The beta-coefficient from the regression was exponentiated which approximates to the percentage difference. The P-value for the regression is presented and no associations reached statistical significance. The recessive and dominant models, defined according to the risk allele, are presented in **Supplementary Table S2**.

### Effect of FFAR1 polymorphisms on plasma lipids

There was a nominally significant association (unadjusted) of carrying the G allele for rs2301151 with higher total cholesterol (0.2 mmol/L per G risk allele, **Table 4**). There was a recessive effect of the G allele which was associated with lower (∼23%, P = 0.004, unadjusted) plasma non-esterified fatty acids (NEFA) (**Table S3**). There was a recessive effect of the common G allele of rs16970264 on total and LDL cholesterol (**Table S3**). However, carriage of the G allele was protective for HDL cholesterol (0.09 mmol/L higher per G allele (P<0.05, unadjusted)) and there was no effect on the total∶HDL cholesterol (TC∶HDL) ratio (**Table 4**). None of these associations were statistically significant when accounting for multiple comparisons by FDR procedure. There was no effect of rs1573611 on lipid measures.

**Table 4:** Fasting plasma lipids by genotype for three SNPs of *FFAR1*.

	rs2301151					rs1573611					rs16970264				
				Difference	P				Difference	P				Difference	P
	AA	GA	GG			TT	TC	CC			AA	GA	GG		
***Total Cholesterol (mmol/L)***															
mean	5.57	5.77	6.14	0.2	0.01	5.62	5.59	5.71	0.08	0.33	5.67	5.39	5.72	0.3	0.02
SE	0.06	0.07	0.25			0.20	0.01	0.06			0.68	0.14	0.05		
n	233	155	16			20	137	247			3	47	349		
***LDL Cholesterol (mmol/L)***															
mean	3.51	3.62	3.9	0.13	0.09	3.59	3.5	3.6	0.05	0.50	4.02	3.31	3.61	0.23	0.05
SE	0.06	0.07	0.22			0.19	0.08	0.05			0.63	0.13	0.05		
n	233	155	16			20	138	247			3	47	350		
***HDL Cholesterol (mmol/L)***															
mean	1.41	1.44	1.60	0.04	0.16	1.40	1.40	1.45	0.04	0.17	1.15	1.36	1.44	0.09	0.05
SE	0.02	0.03	0.08			0.08	0.03	0.02			0.10	0.05	0.02		
n	233	155	16			20	137	247			3	47	349		
***Triglycerides (mmol/L)***															
Geometric mean	1.27	1.35	1.34	1.04	0.35	1.23	1.32	1.30	0.99	0.75	1.10	1.42	1.29	0.95	0.49
95% CI	[1.20, 1.36]	[1.25, 1.46]	[1.13, 1.59]			[0.98, 1.55]	[1.23, 1.43]	[1.22, 1.39			[0.71, 1.69]	[1.21, 1.67]	[1.22, 1.35]		
n	233	154	16			20	137	246			3	46	349		
***Non-esterified fatty acids (µmol/L)***															
Geometric mean	651	650	522	0.94	0.04	630	648	646	0.99	0.63	603	635	650	1.04	0.5
95% CI	[622, 681]	[610, 693]	[397, 689]			[528, 752]	[611, 687]	[614, 679]			[151, 2403]	[563, 716]	[624, 676]		
n	226	149	16			20	132	240			3	46	337		
***Total: HDL cholesterol ratio***															
mean	4.14	4.25	3.98	0.05	0.60	4.22	4.24	4.15	−0.08	0.36	4.94	4.14	4.17	−0.02	0.91
SE	0.07	0.09	0.23			0.23	0.10	0.07			0.41	0.16	0.06		
n	233	155	16			20	137	247			3	47	349		

Data are presented as mean, SEM, n (Total, LDL, HDL and total: HDL cholesterol) or geometric mean, 95% confidence intervals, n (triglycerides, non-esterified fatty acids) stratified by genotype for each of the three SNPs. For each genotype the risk allele was defined as the BMI-increasing allele except for rs16970264 where the risk allele was defined according to the total-cholesterol increasing effect and the data are presented as the additive model. The differences in trait by genotype were assessed by linear regression analysis coding the number of risk alleles as 0,1,2. Data for plasma NEFA and triglycerides were logged for regression analysis. The beta-coefficient from the regression was exponentiated which approximates to the percentage difference. The P-value for the regression is presented. No associations reached statistical significance by the FDR-controlling procedure q* = 0.05. The recessive and dominant models, defined according to the risk allele are presented in **Supplementary Table S3**.

**Figure 2:**
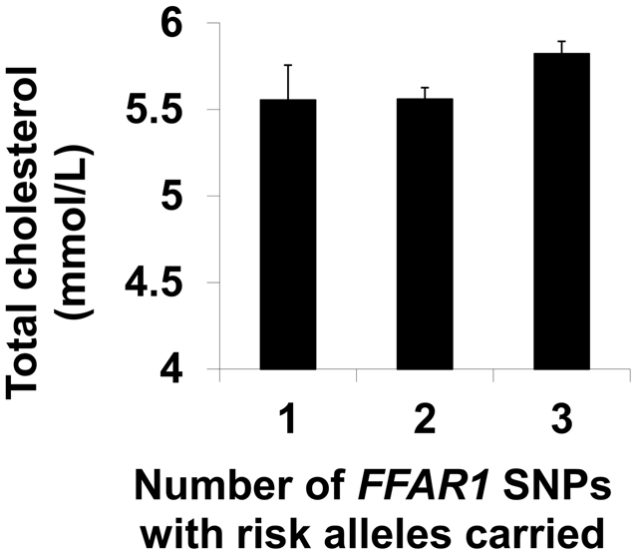
The cumulative effect of carrying risk alleles in three SNPs of *FFAR1* on total cholesterol. Data presented are mean ± SEM of total cholesterol for individuals according to the number of SNPs for which risk alleles were present. Each SNP in the *FFAR1* region which was examined (rs2301151; rs1573611; rs16970264) was scored 0 or 1 based on the presence of risk alleles (dominant model). The number of SNPs with risk alleles was summed. All individuals had at least 1 SNP with risk alleles present. There was a cumulative effect on total cholesterol of 0.18±0.08 mmol/L per SNP, P = 0.03 assessed by linear regression analysis with age, gender and BMI. This was not statistically significant when accounting for multiple comparisons by the Benjamini and Hochberg False Discovery Rate procedure with q* = 0.05.

There was a cumulative effect of the number of SNPs for which risk alleles were carried, on total plasma cholesterol (**Figure 2**). For an increasing number of SNPs where at least one risk allele was carried, the total cholesterol was higher (effect = 0.18±0.08 mmol/L per SNP, P = 0.03), however this was not statistically significant when accounting for multiple comparisons. There was no significant SNP×SNP interaction effect examined as either a two-way or three-way interaction using the additive model. There was no evidence of an effect of change in plasma fatty acid profile integrating with the cumulative number of SNPs for which at least 1 risk allele was carried, to have a significant effect on change in or final level of any lipid parameters.

## Discussion

The key findings of the present study were that SNPs of the *FFAR1* gene region were associated with cumulative differences in BMI, body composition and total cholesterol in the three SNPs studied, although the effects on total cholesterol were not significant after accounting for multiple comparisons.

The predominant adverse effects on BMI and body fat were mediated by carrying the G allele of rs2301151 and the C allele of rs1573611, with SNP rs1573611 also showing gender specific effects of carrying the C-allele, with an increased waist circumference in females only. The predominant adverse effects on plasma lipids were mediated by the G allele of rs2301151 and the G allele (which was the common allele) of rs16970264. However, the associations with lipids did not remain significant when accounting for multiple comparisons.

SNP rs2301151 is in the coding region of the *FFAR1* gene and results in a non-synonymous substitution (Arg211His) located in the intracellular region between transmembrane 5 domain and transmembrane 6 domain of the 7 transmembrane domain protein, [12]. The other SNPs (rs1573611 and rs16970264) are in the non-coding region of gene *FFAR1*, in this regard we are assuming that these SNPs affect *FFAR1* as the closest gene, but cannot exclude the possibility that the observed effects were mediated by another locus in LD with one or other of these SNPs (see **Figure S1**).

There is no defined metabolic pathway that links FFAR1 with body weight. However, FFAR1 receptor is found throughout the CNS [1], and therefore it may play a role in appetite regulation. Others have hypothesised that FFAR1 may be the receptor that coordinates the appetite suppression in response to FFA [6], [13], [14], [15], and that FFAR1 has a role in the taste perception of fat [8]. If variation in this gene is associated with changes in the latter, it is possible this could exert subtle changes in energy homeostasis. Since *FFAR1* has been linked to beta-cell function and type 2 diabetes [2], [3], [7], it is possible that changes in insulin metabolism could impact on energy homeostasis and consequently BMI and body composition. A rare variant, the Gly180Ser mutation, was previously shown to increase in frequency with increasing BMI, providing further support for linkage between variants of the *FFAR1* gene and BMI reported in the present study. Vettor *et al.* [6] suggested FFAR1 may provide a hypothalamic link between the sensing of adequate circulating fatty acid levels and subsequent regulation of energy intake.

The BMI-increasing G allele of rs2301151 was also associated with higher total cholesterol, which was maintained when accounting for the *FFAR1*-associated changes in BMI. SNP rs16970264 also appeared to modulate blood lipids with the most common GG genotype being associated with higher total and LDL cholesterol, but also being associated with higher, protective levels of HDL cholesterol. However these exploratory findings did not reach significance when accounting for multiple comparisons and would require further investigation in an independent cohort. Interestingly, *FFAR1* ^−/−^ animals are somewhat protected against the effects of a high fat diet (HFD), with reduced hyperinsulinaemia, glucose-intolerance or insulin-resistance compared to WT mice [5], and without the increases in hepatic steatosis, plasma triacylglycerol or hepatic glucose output seen in the WT mice [5]. Therefore in rodents, FFAR1 mediates metabolic responses to dietary fats. An oral lipid tolerance test was previously found to be associated with suppressed insulin and increased glucose responses in carriers of the rare Gly180Ser mutation, also implicating *FFAR1* in lipid handling in humans [6]. Changes in the handling of triacylglycerol can have effects on lipoprotein metabolism. An interesting finding was fasting plasma NEFA levels were also lower (∼77% of AA/AG genotypes, P = 0.004) in subjects who were homozygous for the G allele of rs2301151 (**Supplementary Table S3**). It is possible that there is an effect on lipid handling at the adipocyte with variation in the *FFAR1* gene. Although at a lower level, *FFAR1* is expressed in the adipocyte. A recent report suggests that the receptor is present in omental adipose tissue, a key regulator of insulin sensitivity. Genes closely related to *FFAR1* are *FFAR2* and *FFAR3* which both suppress FFA output from the adipocyte when stimulated. Recent reports suggest that there is CNS regulation of adipose tissue metabolism. It could be that the *FFAR1* SNPs may change the central signalling to adipocyte reducing FFA output and enhancing beta cell function further. None of the SNPs are in high LD (r^2^>0.8, supplementary Figure S1) with SNPs of nearby *FFAR* genes, however SNP rs1573611 is in moderate LD (r^2^>0.66) with a SNP from the *FFAR3* gene. Given this, and the proximity of the genes in this cluster, it is possible these SNPs affect the function of neighbouring *FFAR* genes.

Since *FFAR1* is predominantly expressed in the pancreas, and to a lesser extent in the brain, it is reasonable to expect polymorphisms in this receptor to exert greater effects on beta-cell function. The GG allele of rs2301151 was associated with a nominally higher disposition index (DI) with a trend (P = 0.06) towards a higher first phase/acute insulin response (AIRg, **Supplementary Table S2**). It is of interest that there appears to be an interaction between an increase in the number of risk alleles carried, and the change in the receptor agonists (plasma fatty acid greater than C-6) on acute insulin response (AIRg)DI, suggesting a gene-diet interaction, although we did not have sufficient power in this study to formally assess a gene-diet interaction. As there was no difference in insulin sensitivity (Si) or fasting glucose, it appears that this genotype may be associated with a higher insulin secretion for an equivalent degree of insulin sensitivity, ultimately stressing the pancreas. However, these effects were only seen when assessed as a recessive model with a small number of subjects (n = 14) with the GG genotype, so these results should be viewed with caution. SNP rs2301151 investigated in the current study corresponds with the Arg211His polymorphism, which had been found previously to have no effect on glucose or insulin responses to an oral glucose challenge [6]. However, in the study of Vettor *et al.* [6] there was also no difference in BMI or lipids by genotype, this may have been due to a much higher average BMI, and subsequent differences in metabolic profile in that cohort compared to our cohort (mean BMI ∼37 kg/m^2^ cf ∼28 in our cohort). Although we were unable to successfully genotype the SNP rs1978013, which had been found previously to be associated with beta-cell function [7], there was moderate linkage disequilibrium with the more common rs1573611 (r^2^ = 0.31), which was successfully genotyped in the current study. There was no association of this SNP with measures of insulin sensitivity or beta cell function, except for fasting glucose which was lower (−0.31 mmol/L, P = 0.02) in the TT genotype, and was also associated with increased BMI, waist circumference and body fat percent. The protective effects on fasting glucose appear contradictory to the effects on BMI, however the number of subjects with this genotype was 20 (out of a total 404) so these results may be difficult to interpret due to low subject numbers.

There have been very few human studies on the effects of variation in the *FFAR1* gene region on metabolic phenotypes. The differences in BMI, body composition and lipids shown in our study of overweight subjects identified to be at increased cardiometabolic risk, were not accompanied by convincing changes in insulin sensitivity or beta cell function. Therefore it appeared that the differences in body composition and lipids were mediated by mechanisms independent of differences in beta-cell function. It is possible that our methods used for measuring insulin sensitivity and beta-cell function were not sensitive enough to detect differences by genotype, however this method was found to be sensitive for detecting changes in insulin sensitivity associated with a small change in weight in these subjects [9]. SNPs in the *FFAR1* region have not previously been identified in lipid or BMI associated GWA studies. In a recent meta-analysis of lipid-associated SNPs there were no regions of high association near the *FFAR1* gene [16]. This is not unexpected, as previously identified candidate genes have often failed to be identified in GWA studies. In this study we concentrated on variants with a minor allele frequency >0.5 with the intention of studying common variants of this gene. Even with this intention the number of subjects homozygous for the SNPs studied was still small which meant the results were interpreted with some caution. Although not significant, there was a predominant percentage of females, compared to males, who were homozygous for the risk (G) allele of rs2301151. Although gender was included as a covariate for all analyses, it is possible that this gender bias could influence the associations.

The effect of change in dietary fatty acids was assessed by using the data on plasma fatty acids profile which is reflective of dietary intake and reflects the receptor environment. The major agonists of the FFAR1 receptor are fatty acids with a chain length greater than 6. Recent in vitro data suggests the n-3 fatty acids show a greater affinity for the receptor. We were unable to show any relationship between increase in plasma fatty acids and any outcome other than acute insulin response (AIRg) at 24 weeks discussed above.

In summary, we demonstrated that three common SNPs of the *FFAR1* gene were associated with body composition and lipid traits. Furthermore, the effects of the 3 SNPs were cumulative on BMI, body fat and total cholesterol. Despite the strong expression of *FFAR1* in the pancreas, these differences appeared to be independent of changes in insulin and beta cell function.

## Supporting Information

Figure S1 Linkage disequilibrium (LD) plot for the *FFAR* gene cluster. This region includes *FFAR1*, *FFAR3* and the pseudogene *GPR42*, with the gene *FFAR2* located 78.5 KB downstream of *GPR42*. LD is expressed as r^2^ values. This plot was generated in Haploview 4.2. From Hapmap download version 3, release R2.
(TIF)

Table S1 BMI, waist circumference and body fat by genotype for recessive and dominant models of three SNPs of *FFAR1*.
(DOC)

Table S2 Measures of insulin sensitivity and beta-cell function by genotype for recessive and dominant models for three SNPs of *FFAR1*.
(DOC)

Table S3 Fasting plasma lipids by genotype for recessive and dominant models for three SNPs of *FFAR1*.
(DOC)
