# Supplementary figures and images for: Variation in the FFAR1 Gene Modifies BMI, Body Composition and Beta-Cell Function in Overweight Subjects: An Exploratory Analysis
Source: PLoS One. 2011 Apr 28;6(4):e19146. doi: 10.1371/journal.pone.0019146 (PMC3084254; doi:10.1371/journal.pone.0019146)

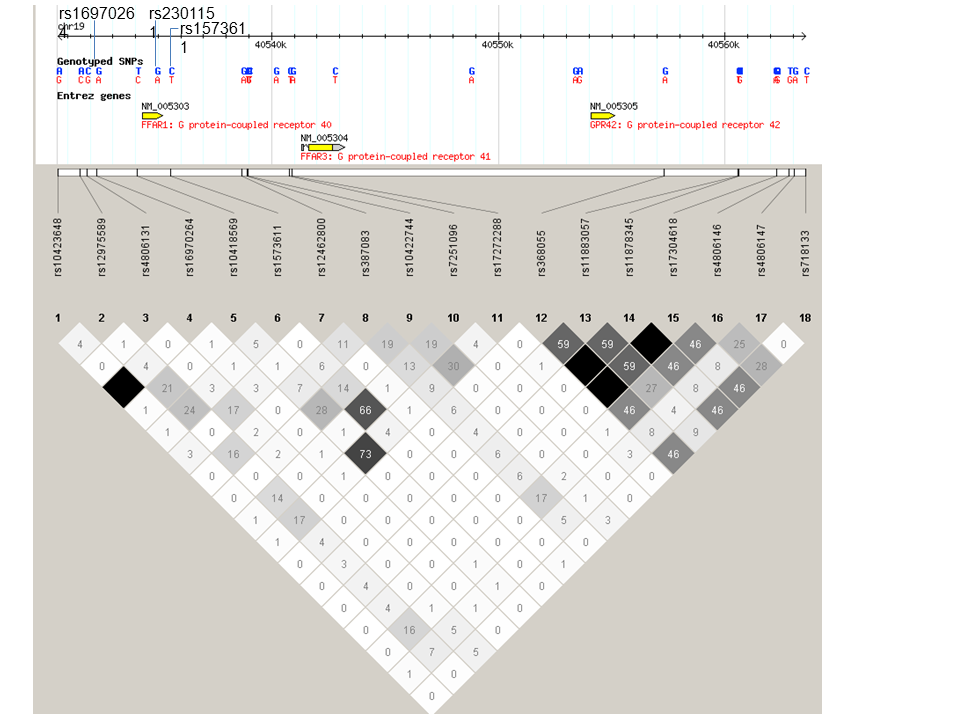

Supplement: Figure S1 — Linkage disequilibrium (LD) plot for the FFAR gene cluster. This region includes FFAR1, FFAR3 and the pseudogene GPR42, with the gene FFAR2 located 78.5 KB downstream of GPR42. LD is expressed as r2 values. This plot was generated in Haploview 4.2. From Hapmap download version 3, release R2. (TIF) [file pone.0019146.s001.tif]
