# Supplementary material for: Variation in the FFAR1 Gene Modifies BMI, Body Composition and Beta-Cell Function in Overweight Subjects: An Exploratory Analysis
Source: PLoS One. 2011 Apr 28;6(4):e19146. doi: 10.1371/journal.pone.0019146 (PMC3084254; doi:10.1371/journal.pone.0019146)
Supplement: Table S1 — BMI, waist circumference and body fat by genotype for recessive and dominant models of three SNPs of FFAR1. (DOC) [file pone.0019146.s002.doc]

**Table S1.** BMI, waist circumference and body fat by genotype for recessive and dominant models of three SNPs of *FFAR1*

Data are presented as mean, SEM, n stratified by genotype for each of the three SNPs. For each genotype the risk allele was defined as the BMI-increasing allele and the data are presented as the recessive and dominant models according to the risk allele. The differences in trait by genotype were assessed by linear regression analysis coding the number of risk alleles as 0,1-with 1 homozygous for the risk allele for the recessive model, and 1 for heterozygotes and homozygous for the risk allele in the dominant model. The P-value for the regression is presented and is in bold when reaching significance by the FDR-controlling procedure q*=0.05. The additive model is presented in **Table 2.**
