# Supplementary material for: Variation in the FFAR1 Gene Modifies BMI, Body Composition and Beta-Cell Function in Overweight Subjects: An Exploratory Analysis
Source: PLoS One. 2011 Apr 28;6(4):e19146. doi: 10.1371/journal.pone.0019146 (PMC3084254; doi:10.1371/journal.pone.0019146)
Supplement: Table S3 — Fasting plasma lipids by genotype for recessive and dominant models for three SNPs of FFAR1. (DOC) [file pone.0019146.s004.doc]

**Table S3.** Fasting plasma lipids by genotype for recessive and dominant models for three SNPs of *FFAR1*


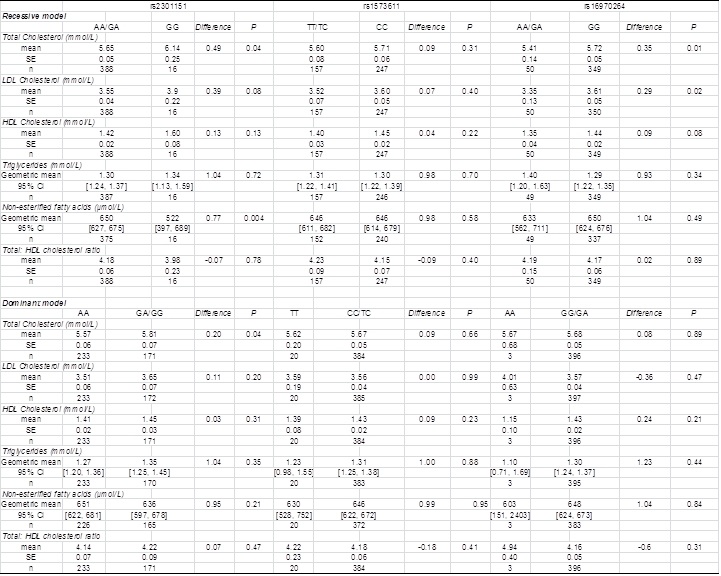


Data are presented as mean, SEM, n or geometric mean, 95% confidence intervals, n (triglycerides, non-esterified fatty acids) stratified by genotype for each of the three SNPs. For each genotype the risk allele was defined as the BMI-increasing allele except for rs16970264 where the risk allele was defined according to the total-cholesterol increasing effect and the data are presented as the recessive and dominant models. The differences in trait by genotype were assessed by linear regression analysis coding the number of risk alleles as 0,1-with 1 homozygous for the risk allele for the recessive model, and 1 for heterozygotes and homozygous for the risk allele in the dominant model. Data for NEFA and triglycerides were logged for regression analysis. The beta-coefficient from the regression was exponentiated which approximates to the percentage difference. The P-value for the regression is presented. No associations were significant when accounting for multiple testing. The additive models are presented in **Table 4**.
